# Ictal High-Frequency Oscillation for Lateralizing Patients With Suspected Bitemporal Epilepsy Using Wavelet Transform and Granger Causality Analysis

**DOI:** 10.3389/fninf.2019.00044

**Published:** 2019-06-28

**Authors:** Tao Han, Zhexue Xu, Jialin Du, Qilin Zhou, Tao Yu, Chunyan Liu, Yuping Wang

**Affiliations:** ^1^Department of Neurology, Xuanwu Hospital, Capital Medical University, Beijing, China; ^2^Beijing Key Laboratory of Neuromodulation, Beijing, China; ^3^Center of Epilepsy, Beijing Institute for Brain Disorders, Capital Medical University, Beijing, China; ^4^Department of Functional Neurosurgery, Xuanwu Hospital, Capital Medical University, Beijing, China

**Keywords:** bilateral temporal epilepsy, localization, lateralization, wavelet transform, Granger causality

## Abstract

Identifying lateralization of bilateral temporal lobe epilepsy (TLE) is a challenging issue; scalp electroencephalography (EEG) and routine band electrocorticography (ECoG) fail to reveal the epileptogenic focus for further temporal lobectomy treatment. High-frequency oscillations (HFOs) can be utilized as a biomarker for lateralizing the onset zone in suspected bitemporal epilepsy. Except subjective vision detect the HFOs, objective verification should be performed to raise the accuracy. In the present research, we prospectively studied 10 patients with refractory temporal seizures and who underwent ECoG with wide-band frequency amplifiers (2,048 Hz); all patients had a class I outcome after temporal resection. Pre- and ictal HFOs will be analyzed by wavelet transform (WT) and Granger causality (GC) to objectively verify lateralization of the seizure onset zone (SOZ). WT analysis showed ictal HFOs in 10 patients mainly covered from 80 to 115 Hz (average, 92.59 ± 10.23 Hz), and there was distinct bandpass boundary between pre-ictal HFOs and ictal HFOs. GC analysis showed five patients (2, 4, 5, 6, and 7), no matter the pre-ictal or ictal state, had the highest GC degree in SOZ itself. The remaining patients (1, 3, 8, 9, and 10) had the highest GC degree in SOZ with its adjacent regions in the pre-ictal and ictal stages. GC analysis further confirmed the result of the WT and suggested HFOs are initiated and propagated in the local brain region mainly, afterward, transmitting to adjacent brain regions. These results indicated that the combination of WT and GC analyses significantly contributes to accurate lateralization in patients with suspected bitemporal epilepsy.

## Introduction

Epilepsy is a chronic neurological disorder manifested by abnormal excessive or synchronous neuronal activity in the brain. It affects more than 50 million people worldwide (WHO, 2018). Although most patients could achieve seizure control with antiepileptic drug (AED) application, unfortunately, despite more appropriate AED therapy, approximately 30% of patients still experience recurrent seizures (de Tisi et al., 2011; Kwan et al., 2011; Barr and Morrison, 2015). Temporal lobe epilepsy (TLE) is the most common type of partial epilepsy often refractory to AEDs and referred for epilepsy surgery (Téllez-Zenteno and Hernández-Ronquillo, 2012). Surgical removal of brain tissue involved in the seizure onset generation for TLE is an effective treatment (Schomer and Lewis, 2012) that could benefit nearly 70% of patients with TLE (de Tisi et al., 2011; Sherman et al., 2011). So, accurate lateralization is crucial before temporal lobectomy.

There is a big challenge in lateralizing bilateral TLE; this is due to conventional scalp electroencephalography (EEG) and magnetic resonance imaging (MRI) often being non-lateralized and discordant in ictal localization. In order to overcome the limitations, intracranial electrodes are often implanted to confirm the origin of the seizures. Routine band invasive EEG monitoring fails to identify the seizure laterality. Recently, high-frequency oscillations (HFOs) have been widely recognized as a biomarker for the epileptic zone (Jacobs et al., 2012; Maeike et al., 2012; Dümpelmann et al., 2015). They are grouped into ripples (80–250 Hz) and fast ripples (250–500 Hz) and have been associated to seizure genesis (Staba et al., 2002; Urrestarazu et al., 2007; Bragin et al., 2015). Removal of brain regions with HFOs seems to result in favorable surgical outcome, and the ratio between ripple rates in removed and nonremoved contacts was significantly higher in patients with a favorable outcome [International League Against Epilepsy (ILAE) classes 1–3] compared to patients with a poor outcome (ILAE classes 4–6; Julia et al., 2010). To some extent, HFOs are better biomarker than others in lateralizing seizure origin in bilateral TLE. Our previous study had subjectively evaluated the value of HFOs in lateralizing bitemporal epilepsy (Liu et al., 2016); objectively investigating HFOs are not performed yet.

Generally, EEG signal is nonstationary, the time–frequency domain, like wavelet transform (WT; Gadhoumi et al., 2012), provides higher success than signal features that were extracted in the time or frequency domain; furthermore, it has been adopted in automatic seizure detection (Ayoubian et al., 2013). Thus, it is significantly utilized in detecting the power of HFOs. One approved viewpoint is seizures are thought to spatially initiate and propagate from a discrete seizure focus (Bertram et al., 1998), and whether the HFOs also initiate and propagate in unilateral temporal lobe and whether these characteristics contribute more to lateralizing seizure focus remain unrevealed. An animal model of TLE research highlights the utility of Granger causality (GC) to reveal dynamic directional temporal relationships between multichannel local field potential (LFP) recordings and indicated distinct patterns of directional GC relationships within the hippocampus prior to and during seizure onset (Cadotte et al., 2010). Two cases of focal seizure disorder patients were analyzed by GC to measure causality across brain regions involved in ictal events, and it was found that both examples have shown hypercoupling near the seizure foci and low causality across nearby brain regions (Coben and Mohammad-Rezazadeh, 2015). So, using GC analysis to reveal directional relationships may be more helpful to identify the seizure onset zones (SOZs). We postulate that WT could objectively reveal HFOs, and GC analysis not only strengthens the accuracy rate in lateralizing bilateral TLE but discovers propagating regularity.

## Materials and Methods

### Patient Selection

Ten patients clinically suspected to have bitemporal epilepsy and who were undergoing investigation for their epilepsies with intracranial electrode implantation enrolled at Beijing Xuanwu Hospital Comprehensive Epilepsy Center between April 2012 and April 2014 (the subjects in this study partly overlapped with those in our previous work). Patients had a comprehensive noninvasive evaluation prior to intracranial exploration, and sites for electrode placement were individualized based on seizure semiology, clinical history, and previous electrophysiological investigations; implanted electrodes are given in Table 1. Basic MRI scanning was performed on all patients using a Siemens Trios 3-T scanner (Siemens, Erlangen, Germany) with conventional epilepsy protocols, including T1WI, T2WI, T2-FLAIR, and oblique coronal T2-FLAIR. Additionally, ordinary whole-brain volumetric series were obtained by magnetization-prepared rapid gradient echo (MPRAGE) sequence, and T2-FLAIR oblique coronal images of both hippocampi were also acquired from perpendicular to the long axis. Few patients were scanned by single photon emission computed tomography (SPECT), magnetic resonance spectroscopy (MRS), and magnetoencephalography (MEG). All patients gave written informed consent and the study was approved by the Medical Research Ethics Committee at Xuanwu Hospital Capital Medical University.

**Table 1:** Number of channels analyzed out of the total bipolar channels recorded, and description of the sites of electrode insertion.

Patient	Channels analyzed/recorded	Ictal HFOs recorded times	Places of electrodes
1	45/48	1	LH, LTB, LTBb, RH, RTB, RTBb
2	16/16	4	LTB, RTB
3	38/38	4	LH, LTP, LTB, RH, RTP, RTB
4	32/32	1	LTP, LTB, RTP, RTB
5	28/28	1	LH, LTB, RH, RTB
6	23/24	1	LH, LTP, RH, RTP
7	25/40	1	LH, LTP, LTB, RH, RTP, RTB
8	44/46	3	LH, LTP, LTB, RH, RTP, RTB
9	40/40	2	LH, LTP, LTB, RH, RTP, RTB
10	39/40	2	LH, LTP, LTB, RH, RTP, RTB

*LH, left hippocampus; LTP, left temporal polar; LTB, left temporal-basal region; LTBb, left bottom of temporal-basal region; RH, right hippocampus; RTP, right temporal polar; RTB, right temporal-basal region; RTBb, right bottom of temporal-basal region*.

### High Sampling Data Recordings

Bilateral strip electrodes with four to eight one-sided circular contacts (2.3 mm in diameter and with a center-to-center separation of 10 mm) were placed over the temporal pole and/or temporal basal region in all 10 patients; effective surface area was 4.15 mm^2^. Furthermore, additional depth electrodes were placed into the mesial temporal lobe structures in eight patients *via* the occipitotemporal (Blatt et al., 1997; Van Roost et al., 1998; Kral et al., 2002); it was composed of four to six cylindrical contacts (2.3 mm long, 1 mm in diameter, 10 mm apart center to center) that were mounted on a 1-mm-wide flexible plastic probe. Its effective surface was 7.2 mm^2^. Electrode position was confirmed with postoperative cranial x-rays, fine-cut computed tomography (3-mm cuts), and MRI. Electrocorticography (ECoG) data were acquired in a 128-channel Micromed system (16 bit, bandwidth at 3 dB: 0.5–100 Hz, Mogliano Veneto, Italy) by conventional sampling rate of 512 Hz and higher sampling rate of 2,048 Hz using a 256-channel broadband frequency amplifier system (16 bit, bandwidth at 4 dB: 0.1–500 Hz, Yunshen Technology Limited Company, China).

The electrode/contact least likely to be involved in seizure onset and with the least artifacts was selected as a reference. Seizure onset was defined as earliest occurrence of rhythmic sinusoidal activity or repetitive spikes that clearly were distinctive from the background and evolved in frequency and morphology (Modur et al., 2011). The ictal onset zone was defined as the contacts that showed the seizure onset alteration in invasive EEG (iEEG). The iEEG was recorded using an input filter of 0.5–100 Hz and a sensitivity of 500–1,000 μV/cm. HFOs were filtered as frequencies >80 Hz with a root mean square amplitude increase of more than five times the standard deviation compared to the background EEG (Bragin et al., 2015), to observe the evolution of the HFOs in the pre-ictal and ictal periods.

### Marking Ictal High-Frequency Oscillations

Two senior neurologists from Beijing Xuanwu Hospital Comprehensive Epilepsy Center confirmed the HFOs that were recorded during the period of implantation. Any HFOs were excluded if they were not associated with the ictal event. Electrodes with poor contact were also excluded. For identifying HFOs, channels were displayed with the maximum time resolution of the computer monitor (0.6 s, 1,200 samples of a signal sampled at 2,000 Hz). The amplitude scale was 1 μV/mm. Characteristic HFOs were chosen visually from unfiltered EEG signals and viewed at 10 s/page in a bipolar montage wherein consecutive contacts on each electrode are compared.

### Wavelet Transform and Granger Causality Analyses

Raw data will be preprocessed before the WT and GC in a brainstorm software^1^ (Tadel et al., 2011). In order to get distinct HFOs, according to the definition of ripples, the bandpass will be set 80–250 Hz, and the time scope will be set 5 s pre- and ictal HFOs. The WT of the ECoG was calculated as:

$$W_{a,b}=\int_{-\infty }^{+\infty }f(t)\frac{1}{\sqrt{\left|a\right|}}\Psi *\left(\frac{t-b}{a}\right)\text{d}t$$

As the scale and translation parameters *a* and *b* are taken at discrete values, discrete WT is obtained. The parameters *a* and *b* are often based on powers of two and called dyadic scales and translations:

$$a_{j}=2^{j},b_{j,k}=k2^{j}\text{ for all, }j\text{, }k\;\in \;Z$$

So the equation becomes:

$$\Psi_{j,k}(t)=2^{-j/2}\Psi (2^{-j}\cdot t-k)\text{ for all, }j,k\in Z$$

The set of *Ψ*_*j,k*_ (*t*) forms a basis of square integrable space *L*^2^ (*R*).

If the basis function *Ψ*_*j,k*_ (*t*) is orthogonal, then the original signal can be reconstructed from the resulting wavelet coefficients accurately and efficiently without any loss of information.

GC methods make use of the variance of prediction errors to extrapolate directional relationships. *X*_1_(*t*) and *X*_2_(*t*) and future values of *X*_1_(*t*) are going to be predicted by using two different data sets: using only the past values of *X*_1_(*t*) and by incorporation of past values of *X*_1_(*t*) and *X*_2_(*t*). If incorporating the past knowledge of *X*_2_(*t*) permits more accurate prediction of *X*_1_, then *X*_2_ could be called a casual to *X*_1_ (Cadotte et al., 2008). Suppose *X*_1_ and *X*_2_ can be represented by single-variable autoregressive models, its basic formulae are as follows:

$$X_{1}(t)=\sum_{j=1}^{m}a_{j}X_{1}(t-j)+\varepsilon_{11}(t)$$

$$X_{2}(t)=\sum_{j=1}^{m}b_{j}X_{2}(t-j)+\varepsilon_{22}(t)$$

A joint predictor of *X*_1_(*t*) can be defined as:

$$X_{1}^{*}(t)=\sum_{j=1}^{m}a_{j}^{*}X_{1}(t-j)+\sum_{j=1}^{m}b_{j}^{*}X_{2}(t-j)+\varepsilon_{12}(t)$$

Here, if the variance of prediction error $\delta_{12}^{2}(\varepsilon_{12})$ is less than the variance of $\delta_{11}^{2}(\varepsilon_{11})$, then it is an indication of a causal interaction from *X*_2_(*t*) to *X*_1_(*t*). The magnitude of causality from *X*_2_ to *X*_1_ is defined as $F_{X_{2}\to X_{1}}=\ln\left(\frac{\delta_{12}^{2}}{\delta_{1}^{2}}\right)$; thus, if $\delta_{1}^{2}=\delta_{12}^{2}$, then the magnitude of causality from *X*_2_ to *X*_1_ is zero.

A single electrode or common average referential montage can result in a bad signal on all channels if the reference contains high-frequency artifacts; thus, bipolar montages are adopted in WT analysis. Because GC is to detect connectivity between two channels, a unipolar montage is adopted in GC analysis. The GC threshold was set 10% of the higher ranking of all channels. GC was analyzed separately 2 s prior and 2 s after ictal seizures between every two channels.

### Surgical Procedure, Follow-Up, and Outcome Classification

All surgical procedures were operated by a single neurosurgeon (TY). The resection included a standard temporal lobectomy of the anterior 4.5–5.5 cm of the temporal lobe, sparing the superior temporal gyrus. The amygdale and anterior one-half to two-thirds of the hippocampus were resected and sent for pathological analysis (Kuzniecky et al., 1993). Specimen analysis was performed by a neuropathologist Dr. Piao Yueshan (YSP). The diagnosis of mesial temporal sclerosis was based on the presence of hippocampal neuronal loss and gliosis (Toga and Berard-Badier, 1982). Postoperatively, patients were followed-up by the surgeon (TY). The mean duration of follow-up was 20.1 months (range, 11–32 months; Table 2). Outcome of operation was determined by a mailed questionnaire and confirmed both by structured telephone interviews and by chart reviews. Long-term outcome classification was assessed by the Engel scale (Wieser et al., 2001).

**Table 2:** Clinical characteristics of the suspected bilateral temporal epileptic patients.

Patient	Age/gender	History of TLE (years)	Seizure type (outcome)	Antiepileptic medications	Neuroimaging	Ictal EEG	Ictal ECoG	Ictal HFOs	Surgery	Follow-up (months)	Pathology
1	22/M	18	Complex partial seizures (Eagle 1); GTCS (Eagle 1)	PHT, OXC	MRI, left HS	B	L	LT	LT, LH	24	FCD I
2	35/F	13	Complex partial seizures (Eagle 1); GTCS (Eagle 1)	PB	MRI, left medial temporal cavernous hemangioma;	B	B	LT	LT, LH	32	CH
3	33/M	13	Complex partial seizures (Eagle 1); GTCS (Eagle 1)	VPA, CBZ	MRI, left HS; SEPCT, hypoperfusion in left temporal cortex	B	B	LH	LT, LH	25	HS
4	45/F	28	Complex partial seizures (Eagle 1); GTCS (Eagle 1)	CBZ	MRI, normal; SPECT, hypoperfusion in right temporal cortex; MEG, left temporal cortex	B	L	LT	LT, LH	31	FCD I
5	27/M	18	Complex partial seizures (Eagle 1)	PHT, CBZ	MRI, right HS	B	B	RT	RT, RH	24	FCD I, HS
6	27/F	4	Complex partial seizures (Eagle 1); GTCS (Eagle 1)	CBZ, OXC	MRI, normal	B	L	LH	LT, LH	19	FCD I
7	26/M	5	Complex partial seizures (Eagle 1); GTCS (Eagle 1)	CBZ, TPM	MRI, left HS; SPECT, hypoperfusion in left temporal cortex; MRS, right hippocampus; MEG, left temporal cortex	B	L	LH	LT, LH	11	FCD IIIa, HSIb
8	30/F	14	Complex partial seizures (Eagle 1) GTCS (Eagle 1)	TPM, PHT, VPA	MRI, normal SPECT, hypoperfusion in bilateral temporal cortex MRS, right hippocampus;	B	R	RT	RT, RH	12	FCD IIIa, HS
9	49/F	26	Complex partial seizures (Eagle 1)	OXC	MRI, left HS	B	L	LH	LT, LH	11	FCD IIIa, HS
10	26/M	22	Complex partial seizures (Eagle 1) GTCS (Eagle 1)	PB, CBZ, VPA	MRI, right HA	B	L	LH	LT, LH	12	FCD I

*GTCS, generalized tonic-clonic seizures; PHT, Phenytoin; OXC, oxcarbazepine; CBZ, carbamazepine; VPA, Sodium Valproate; TPM, Topiramate; PB, phenobarbital; CH, cavernous hemangioma; HS, hippocampal sclerosis; HA, hippocampal atrophy; L, left; R, right; B, bilateral; LT, left temporal lobe; RT, right temporal lobe; LH, left hippocampus; RH, right hippocampus; FCD, focal cortical dysplasia*.

## Results

### Patients

Ten patients were included in the study (female/male: 5/5; mean age: 32.00 ± 8.78). The average duration of seizure disorder was 14.20 ± 8.66 years. MRI scanning was performed on all patients; SPECT, MRS, and MEG scanning were performed on only some patients. MRI showed left hippocampal sclerosis (HS) in five (patients 1, 3, 5, 7, and 9), hippocampal atrophy (HA) in one (patient 10), left medial temporal cavernous hemangioma (CH) in one (patient 2), and no clear abnormalities in three patients (patients 4, 6, and 8). SEPCT showed hypoperfusion in the left temporal cortex in two (patients 3 and 7), in the right temporal cortex in one (patient 4), and in the bilateral temporal cortex in one patient (patient 8). The MRS showed abnormal function in the right hippocampus (RH; patients 7 and 8). The MEG showed dipoles in the left hippocampus (LH; patients 4 and 7). Scalp ictal EEG showed that all the patients had bilateral TLE; routine band ECoG showed left TLE in six (patients 1, 4, 6, 7, 9, and 10), right TLE in one (patient 8), and bilateral TLE in three patients (patients 2, 3, and 5). All details are shown in Table 2.

### Wavelet Transform Analysis of Electrocorticography

**Figure 1:**
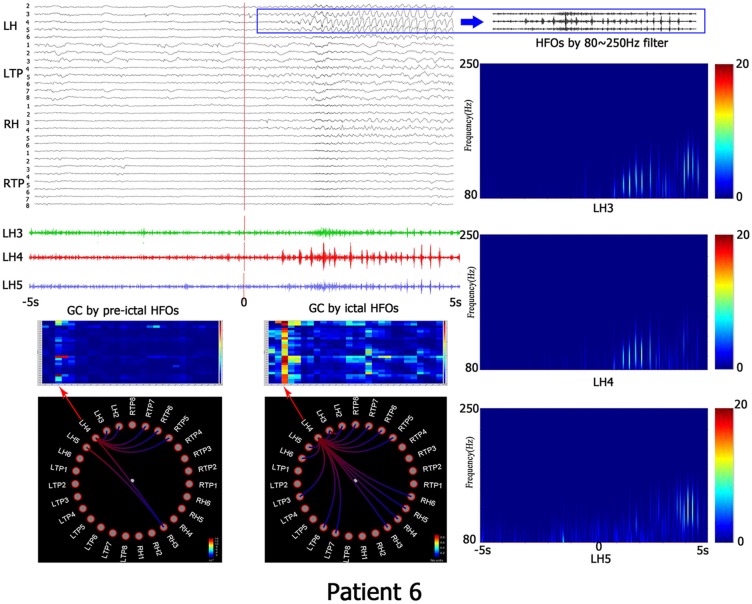
This is the representing result of patient No. 6. All recorded channels are in top left corner, the time scope is 10 s, the red line represents the beginning of ictal HFOs, blue PANE shows the HFOs by 80–250 Hz filter. The left down corner is the matrix and granger causality (GC) figures which indicates the causality information. The right line is the time-frequency analyzed by wavelet transform (WT). LH, left hippocampus; LTP, left temporal polar; RH, right hippocampus; RTP, right temporal polar.

After visually detecting the entire video ECoG of each patient, a total of 20 ECoG segments with HFOs were analyzed in 10 s scope pre- and ictal HFOs. The time–frequency power was calculated by the formula ECoG signal units^2^/Hz · 10^12^. The HFOs in 10 patients covered from 80 Hz to 115 Hz (average, 92.59 ± 10.23 Hz; Table 3). The representative patient result is shown in Figure 1.

**Table 3:** The time-frequency power of each patient (units: Hz).

Segments/patients	1	2	3	4
1	85/90	—	—	—
2	85/95	85/95	85/95	85/95
3	85/90	85/90/100/115	90/95/100	80/85/95
4	80/85	—	—	—
5	90	—	—	—
6	95/100/110	—	—	—
7	80/85/90	—	—	—
8	85/90	85/90	85/90	85/90
9	90/100	100/110	90/100/115	90/110
10	85/90	85/90	—	—

### Granger Causality Analysis of Electrocorticography

**Figure 2:**
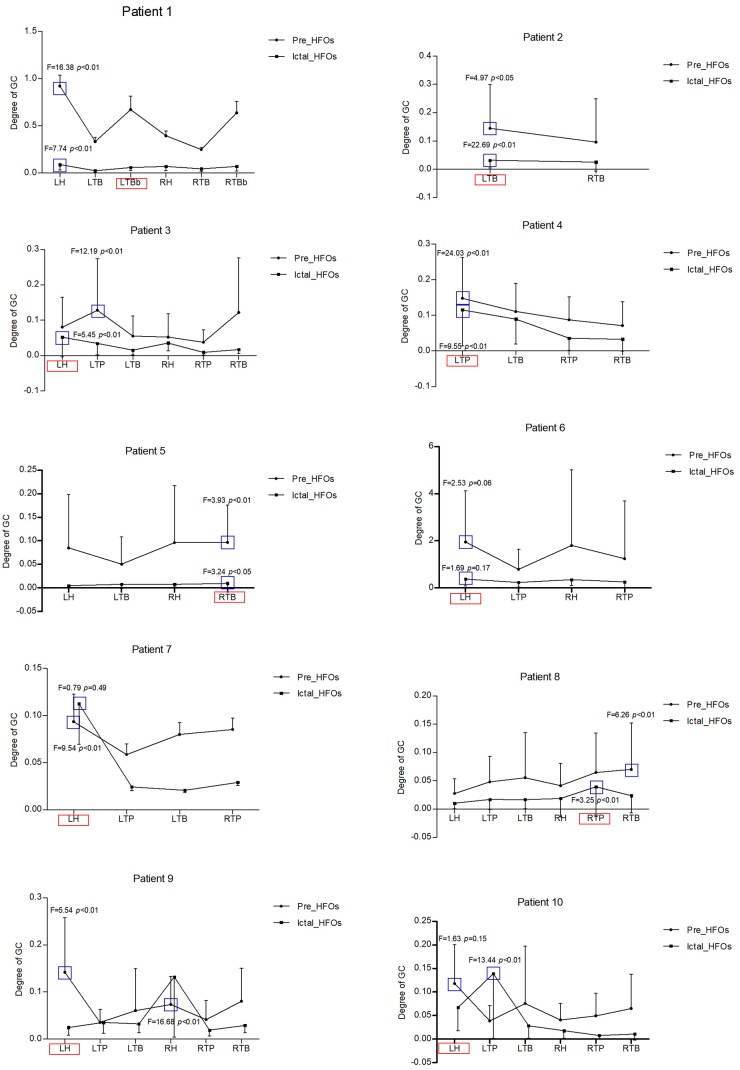
These are all representing results in 10 patients regarding the GC analysis. X-axis is the different brain regions, Y-axis is the degree of GC, the bold circle shows the GC of pre-HFOs, the black square shows the GC of ictal HFOs. The red pane represents the ictal onset region with high-frequency oscillations (HFOs), the blue pane represents the highest GC with ictal onset region among all brain regions with HFOs either in pre-ictal or in ictal state. LH, left hippocampus; LTP, left temporal polar; LTB, left temporal-basal region; LTBb, left bottom of temporal-basal region; RH, right hippocampus; RTP, right temporal polar; RTB, right temporal-basal region; RTBb, right bottom of temporal-basal region.

When the electrode with HFOs was confirmed by WT analysis, causality and connectivity between electrode with HFOs and other electrodes were analyzed by GC. Representing results showed that HFOs originated in the left bottom of the temporal-basal region (LTBb) in patient 1; the LH had the highest causality with LTBb (pre-HFOs, 0.92 ± 0.87; ictal HFOs, 0.09 ± 0.06). HFOs of patient 2 originated in the left temporal-basal region (LTB), and the GC analysis showed the highest causality occurred within the LTB (pre-HFOs, 0.13 ± 0.15; ictal HFOs, 0.05 ± 0.05). Patient 3 showed HFOs originated from the LH, the left temporal polar (LTP) had the biggest GC with LH (pre-HFOs, 0.14 ± 0.15), and LH had the highest causality in the period of ictal HFOs (ictal HFOs, 0.03 ± 0.02). The HFOs of patient 4 originated in the LTP, and the GC analysis showed the highest causality occurred within itself (pre-HFOs, 0.15 ± 0.11; ictal HFOs, 0.12 ± 0.10). The HFOs of patient 5 originated in right temporal-basal region (RTB), and the GC analysis showed the highest causality occurred within itself (pre-HFOs, 0.09 ± 0.06; ictal HFOs, 0.01 ± 0.01). HFOs of patient 6 originated in the LH, and the GC analysis showed the highest causality occurred within itself (pre-HFOs, 1.95 ± 2.18; ictal HFOs, 0.38 ± 0.29). The HFOs of patient 7 originated in the LH, and the GC analysis showed the highest causality occurred within itself (pre-HFOs, 0.09 ± 0.10; ictal HFOs, 0.11 ± 0.15). The HFOs of patient 8 originated in the right temporal polar (RTP), RTB had the highest causality with RTP (pre-HFOs, 0.07 ± 0.08), and RTP had the highest causality within itself in the period of ictal HFOs (ictal HFOs, 0.04 ± 0.05). The HFOs of patient 9 originated in the LH, it had the highest causality within itself (pre-HFOs, 0.14 ± 0.12), and the RH had the highest causality with the LH in the period of ictal HFOs (ictal HFOs, 0.13 ± 0.13). HFOs of patient 10 originated in the LH, it had highest causality within itself (pre-HFOs, 0.12 ± 0.08), and LTP had the highest causality with the LH in the period of ictal HFOs (ictal HFOs, 0.14 ± 0.14). All details are shown in Table 2 and Figure 2.

## Discussion

The present research showed that the frequency of ictal HFOs is around 80–115 Hz by WT analysis, and the GC analysis indicated, no matter the pre- or ictal HFOs, that highest causality between electrodes with HFOs mainly originated from the SOZ and then propagated into adjacent brain regions. Combining the WT and GC analyses is more significant in verifying lateralization of suspect bitemporal epilepsy.

WT is an effective tool in signal processing due to time–frequency localization and multirate filtering (Acharya et al., 2011). These properties can be used to extract the desired local characteristics from an input signal in time and space. High-frequency intracranial EEG researches have increasing evidence indicating HFOs could be biomarkers of the seizure onset region (Jacobs et al., 2008; Khosravani et al., 2009) and play a critical role in epileptogenicity (Jacobs et al., 2009; Mirowski et al., 2009); what’s more, it was also consistent with HS or other lesions observed in the MRI of patients with TLE. Ripple frequency oscillations are increased in the SOZ more frequently than fast ripple frequency oscillations, and ripples display higher amplitude at the transition from the inter-ictal to the ictal state. Therefore, ripples in the ictal period may have more advantages in laterality localization. Thus, using WT methods to analyze the evolution and power changes of ripples in the pre- and ictal periods may contribute to lateralization of TLE. Previous researches (Wang et al., 2011; Gadhoumi et al., 2012) developed a variety of WT analysis methods to predict seizure onset, and all of them got significant results. Unfortunately, those results were not verified in patients by surgery outcome. In the present research, distinct bandpass boundary between pre-ictal HFOs and ictal HFOs was found by WT analysis, and all patients underwent unilateral temporal lobectomy and achieved good surgical results, which were consistent with the laterality of SOZ determined by high-frequency WT analysis.

Channels were found carrying unequal discriminative power between pre-ictal and ictal states; analyzing dynamic characteristics of seizure onset channels may have better discriminability for lateralizing SOZ (David et al., 2008). Thus, based on the results of the time–frequency analysis, GC analysis was performed on causality and connectivity of pre- and ictal HFOs to further verify the SOZ. According to our results, the GC analysis showed HFOs mainly originated in the SOZ; afterward, it propagated into neighboring brain regions. This method has shown similar results to dynamic causal modeling (Murta et al., 2012), directed transfer function (Ge et al., 2007), and partial directed coherence (Chan et al., 2012) methods that have plausible estimates of human seizure propagation pathways; furthermore, it has been consistent with pathways demonstrated by diffusion tensor imaging (DTI; Bhardwaj et al., 2010). Preliminary findings have shown regions of increased connectivity in the regions of the seizure foci in the ictal period (Liao et al., 2010; Maccotta et al., 2013). In mesial temporal lobe seizure studies, previous research indicated the presence of focal HFOs near the time of seizure onset may be close or within the epileptogenic focus by wavelet analysis; this regularity was also uncovered by GC analysis in the present study. The increased connection was found between electrodes in the SOZ and regions proximal to it in the ictal period. The enhancement of local connection may supply the pathophysiological basis about epileptic foci.

## Conclusion

It is significant to lateralize drug-resistant bilateral temporal epilepsy by HFOs. Analyzing ictal HFOs objectively and quantificationally could provide accurate information regarding location of SOZ; what’s more, combining with the GC will substantially improve accuracy. GC analysis further revealed initial focal electrode tightly connected with ictal HFOs and suggested HFOs initiate and propagate in the local brain region, afterward transmitting to the anatomically adjacent brain regions. WT and GC analyses are significant methods for accurately lateralizing patients with suspected bitemporal epilepsy.

## Limitations

In this research, GC analysis was adopted to further lateralize suspected temporal epilepsy. This method was used to quantify directional temporal relationships between financial time series originally, and then it was broadly performed in neuroscience to explore relationships between different brain regions by investigating directed information flow or causality in the brain. Based on this method, there are many analytic techniques developed, such as directed transfer function and partial directed coherence. Both of them are very useful in calculating the information flow in the epilepsy network, but they are still under research. In the present study, we utilize this method, which has been acknowledged by academics, just adding the evidence for HFOs location.

## Author Contributions

TH performed the data collection and analysis. ZX reviewed the literature and drafted the manuscript. JD and QZ participated in data collection. TY participated in data collection and performed the operation. CL and YW designed the study, supervised the initial drafting and critically revised the manuscript.

## Conflict of Interest Statement

The authors declare that the research was conducted in the absence of any commercial or financial relationships that could be construed as a potential conflict of interest.

## Footnotes

^1^https://neuroimage.usc.edu/brainstorm/

## References

[B1] Acharya U. R. Faust O. Sree S. V. Molinari F. Garberoglio R. Suri J. S. (2011). Cost-effective and non-invasive automated benign and malignant thyroid lesion classification in 3D contrast-enhanced ultrasound using combination of wavelets and textures: a class of ThyroScan algorithms. Technol. Cancer Res. Treat. 10, 371–380. 10.7785/tcrt.2012.500214 21728394

[B2] Ayoubian L. Lacoma H. Gotman J. (2013). Automatic seizure detection in SEEG using high frequency activities in wavelet domain. Med. Eng. Phys. 35, 319–328. 10.1016/j.medengphy.2012.05.005 22647836 PMC4490902

[B3] Barr W. B. Morrison C. (2015). Handbook on the Neuropsychology of Epilepsy. New York, NY: Springer.

[B4] Bertram E. H. Zhang D. X. Mangan P. Fountain N. Rempe D. (1998). Functional anatomy of limbic epilepsy: a proposal for central synchronization of a diffusely hyperexcitable network. Epilepsy Res. 32, 194–205. 10.1016/s0920-1211(98)00051-5 9761320

[B5] Bhardwaj R. D. Mahmoodabadi S. Z. Otsubo H. Snead O. C. III. Rutka J. T. Widjaja E. (2010). Diffusion tensor tractography detection of functional pathway for the spread of epileptiform activity between temporal lobe and Rolandic region. Childs Nerv. Syst. 26, 185–190. 10.1007/s00381-009-1017-1 19915854

[B6] Blatt D. R. Roper S. N. Friedman W. A. (1997). Invasive monitoring of limbic epilepsy using stereotactic depth and subdural strip electrodes: surgical technique. Surg. Neurol. 48, 74–79. 10.1016/s0090-3019(96)00277-7 9199690

[B7] Bragin A. Engel J. Jr. Wilson C. L. Fried I. Buzsáki G. (2015). High-frequency oscillations in human brain. Hippocampus 9, 137–142. 10.1002/(SICI)1098-1063(1999)9:2<137::AID-HIPO5>3.0.CO;2-0 10226774

[B9] Cadotte A. J. DeMarse T. B. He P. Ding M. (2008). Causal measures of structure and plasticity in simulated and living neural networks. PLoS One 3:e3355. 10.1371/journal.pone.0003355 18839039 PMC2556387

[B8] Cadotte A. J. DeMarse T. B. Mareci T. H. Parekh M. B. Talathi S. S. Hwang D. U.. (2010). Granger causality relationships between local field potentials in an animal model of temporal lobe epilepsy. J. Neurosci. Methods 189, 121–129. 10.1016/j.jneumeth.2010.03.007 20304005 PMC2867107

[B10] Chan H. L. Tsai Y. T. Wang Y. C. Ju J. H. Chang B. L. Wu T.. (2012). Partial directed coherence analysis of intracranial neural spikes in epilepsy patients. Conf. Proc. IEEE Eng. Med. Biol. Soc. 2012, 5174–5177. 10.1109/embc.2012.6347159 23367094

[B11] Coben R. Mohammad-Rezazadeh I. (2015). Neural connectivity in epilepsy as measured by granger causality. Front. Hum. Neurosci. 9:194. 10.3389/fnhum.2015.00194 26236211 PMC4500918

[B12] David O. Guillemain I. Saillet S. Reyt S. Deransart C. Segebarth C.. (2008). Identifying neural drivers with functional MRI: an electrophysiological validation. PLoS Biol. 6, 2683–2697. 10.1371/journal.pbio.0060315 19108604 PMC2605917

[B19] de Tisi J. Bell G. S. Peacock J. L. McEvoy A. W. Harkness W. F. Sander J. W.. (2011). The long-term outcome of adult epilepsy surgery, patterns of seizure remission, and relapse: a cohort study. Lancet 378, 1388–1395. 10.1016/S0140-6736(11)60890-8 22000136

[B29] Dümpelmann D. Jacobs J. Schulze-Bonhage A. (2015). Temporal and spatial characteristics of high frequency oscillations as a new biomarker in epilepsy. Epilepsia 56, 197–206. 10.1111/epi.12844 25556401

[B14] Gadhoumi K. Lina J. M. Gotman J. (2012). Discriminating preictal and interictal states in patients with temporal lobe epilepsy using wavelet analysis of intracerebral EEG. Clin. Neurophysiol. 123, 1906–1916. 10.1016/j.clinph.2012.03.001 22480601 PMC3654937

[B15] Ge M. Jiang X. Bai Q. Yang S. Gusphyl J. Yan W. (2007). Application of the directed transfer function method to the study of the propagation of epilepsy neural information. Conf. Proc. IEEE Eng. Med. Biol. Soc. 2007, 3266–3269. 10.1109/iembs.2007.4353026 18002692

[B16] Jacobs J. LeVan P. Chander R. Hall J. Dubeau F. Gotman J. (2008). Interictal high-frequency oscillations (80–500 Hz) are an indicator of seizure onset areas independent of spikes in the human epileptic brain. Epilepsia 49, 1893–1907. 10.1111/j.1528-1167.2008.01656.x 18479382 PMC3792077

[B17] Jacobs J. Levan P. Châtillon C. E. Olivier A. Dubeau F. Gotman J. (2009). High frequency oscillations in intracranial EEGs mark epileptogenicity rather than lesion type. Brain 132, 1022–1037. 10.1093/brain/awn351 19297507 PMC3792079

[B18] Jacobs J. Staba R. Asano E. Otsubo H. Wu J. Y. Zijlmans M.. (2012). High-frequency oscillations (HFOs) in clinical epilepsy. Prog. Neurobiol. 98, 302–315. 10.1016/j.pneurobio.2012.03.001 22480752 PMC3674884

[B20] Julia J. Zijlmans M. Zelmann R. Chatillon C. E. Hall J. Olivier A.. (2010). High-frequency electroencephalographic oscillations correlate with outcome of epilepsy surgery. Ann. Neurol. 67, 209–220. 10.1002/ana.21847 20225281 PMC3769290

[B21] Khosravani H. Mehrotra N. Rigby M. Hader W. J. Pinnegar C. R. Pillay N.. (2009). Spatial localization and time-dependant changes of electrographic high frequency oscillations in human temporal lobe epilepsy. Epilepsia 50, 605–616. 10.1111/j.1528-1167.2008.01761.x 18717704

[B22] Kral T. Clusmann H. Urbach J. Schramm J. Elger C. E. Kurthen M.. (2002). Preoperative evaluation for epilepsy surgery (Bonn Algorithm). Zentralbl. Neurochir. 63, 106–110. 10.1055/s-2002-35826 12457335

[B23] Kuzniecky R. Burgard S. Faught E. Morawetz R. Bartolucci A. (1993). Predictive value of magnetic resonance imaging in temporal lobe epilepsy surgery. Arch. Neurol. 50, 65–69. 10.1001/archneur.1993.00540010059018 8418802

[B24] Kwan P. Schachter S. C. Brodie M. J. (2011). Drug-resistant epilepsy. N. Eng. J. Med. 365, 919–926. 10.1056/NEJMra1004418 21899452

[B25] Liao W. Zhang Z. Pan Z. Mantini D. Ding J. Duan X.. (2010). Altered functional connectivity and small-world in mesial temporal lobe epilepsy. PLoS One 5:e8525. 10.1371/journal.pone.0008525 20072616 PMC2799523

[B26] Liu C. Zhang R. Zhang G. Yu T. Tai J. Du W.. (2016). High frequency oscillations for lateralizing suspected bitemporal epilepsy. Epilepsy Res. 127, 233–240. 10.1016/j.eplepsyres.2016.09.006 27639348

[B27] Maccotta L. He B. J. Snyder A. Z. Eisenman L. N. Benzinger T. L. Ances B. M.. (2013). Impaired and facilitated functional networks in temporal lobe epilepsy. Neuroimage Clin. 2, 862–872. 10.1016/j.nicl.2013.06.011 24073391 PMC3777845

[B28] Maeike Z. Jiruska P. Zelmann R. Leijten F. S. Jefferys J. G. Gotman J. (2012). High-frequency oscillations as a new biomarker in epilepsy. Ann. Neurol. 71, 169–178. 10.1002/ana.22548 22367988 PMC3754947

[B30] Mirowski P. Madhavan D. LeCun Y. Kuzniecky R. (2009). Classification of patterns of EEG synchronization for seizure prediction. Clin. Neurophysiol. 120, 1927–1940. 10.1016/j.clinph.2009.09.002 19837629

[B31] Modur P. N. Song Z. Vitaz T. W. (2011). Ictal high-frequency oscillations in neocortical epilepsy: implications for seizure localization and surgical resection. Epilepsia 52, 1792–1801. 10.1111/j.1528-1167.2011.03165.x 21762451 PMC3188690

[B32] Murta T. Leal A. Garrido M. I. Figueiredo P. (2012). Dynamic causal modelling of epileptic seizure propagation pathways: a combined EEG-fMRI study. Neuroimage 62, 1634–1642. 10.1016/j.neuroimage.2012.05.053 22634857 PMC3778869

[B34] Schomer D. L. Lewis R. J. (2012). Stopping seizures early and the surgical epilepsy trial that stopped even earlier. JAMA 307, 966–968. 10.1001/jama.2012.251 22396519

[B35] Sherman E. M. Wiebe S. Fay-McClymont T. B. Tellez-Zenteno J. Metcalfe A. Hernandez-Ronquillo L.. (2011). Neuropsychological outcomes after epilepsy surgery: systematic review and pooled estimates. Epilepsia 52, 857–869. 10.1111/j.1528-1167.2011.03022.x 21426331

[B36] Staba R. J. Wilson C. L. Anatol B. Itzhak F. Engel J. Jr. (2002). Quantitative analysis of high-frequency oscillations (80–500 Hz) recorded in human epileptic hippocampus and entorhinal cortex. J. Neurophysiol. 88, 1743–1752. 10.1152/jn.2002.88.4.1743 12364503

[B37] Tadel F. Baillet S. Mosher J. C. Pantazis D. Leahy R. M. (2011). Brainstorm: a user-friendly application for MEG/EEG analysis. Comput. Intell. Neurosci. 2011:879716. 10.1155/2011/879716 21584256 PMC3090754

[B38] Téllez-Zenteno J. F. Hernández-Ronquillo L. (2012). A review of the epidemiology of temporal lobe epilepsy. Epilepsy Res. Treat. 2012:630853. 10.1155/2012/630853 22957234 PMC3420432

[B39] Toga M. Berard-Badier M. (1982). Pathological findings in temporal lobe epilepsy in man, animals and experimental models. Electroencephalogr. Clin. Neurophysiol. Suppl. 35, 185–193. 6956494

[B13] Urrestarazu E. Chander R. Dubeau F. Gotman J. (2007). Interictal high-frequency oscillations (100–500 Hz) in the intracerebral EEG of epileptic patients. Brain 130, 2354–2366. 10.1093/brain/awm149 17626037

[B33] Van Roost D. Solymosi L. Schramm J. van Oosterwyck B. Elger C. E. (1998). Depth electrode implantation in the length axis of the hippocampus for the presurgical evaluation of medial temporal lobe epilepsy: a computed tomography-based stereotactic insertion technique and its accuracy. Neurosurgery 43, 819–826. 10.1097/00006123-199810000-00059 9766309

[B40] Wang L. Wang C. Fu F. Yu X. Guo H. Xu C.. (2011). Temporal lobe seizure prediction based on a complex Gaussian wavelet. Clin. Neurophysiol. 122, 656–663. 10.1016/j.clinph.2010.09.018 20980197

[B41] WHO (2018). Epilepsy. Available online at: https://www.who.int/en/news-room/fact-sheets/detail/epilepsy

[B42] Wieser H. G. Blume W. T. Fish D. Goldensohn E. Hufnagel A. King D.. (2001). ILAE Commission Report. Proposal for a new classification of outcome with respect to epileptic seizures following epilepsy surgery. Epilepsia 42, 282–286. 10.1046/j.1528-1157.2001.35100.x 11240604

